# Percutaneous coronary intervention for chronic total coronary occlusion: Do. Or do not. There is no try

**DOI:** 10.1007/s12471-020-01531-w

**Published:** 2020-12-15

**Authors:** P. Knaapen, J. P. Henriques, A. Nap, F. Arslan

**Affiliations:** 1 Department of Cardiology, Heart Center, Amsterdam UMC, University of Amsterdam, Amsterdam Cardiovascular Sciences, Amsterdam, The Netherlands; 2 Department of Cardiology, Vivantes Klinikum am Urban, Berlin, Germany

Chronic total coronary occlusion (CTO) represents the final stage of coronary artery disease (CAD) and is documented in as many as one-fifth of patients. Traditionally, CTOs are treated differently than non-occlusive yet obstructive, i.e. ischaemia-inducing, CAD. Despite comparable symptoms and an identical albeit more advanced disease stage, patients with CTOs are less frequently offered percutaneous coronary intervention (PCI) and more often coronary artery bypass grafting (CABG) or optimal medical therapy alone (OMT) [1]. This disparity in treatment strategy is based on two fundamentally flawed concepts.

The first misconception is that angiographically well-developed collaterals prevent myocardial ischaemia. There is currently overwhelming evidence challenging this outdated concept; (almost) all patients display ischaemia in the myocardium subtended by the CTO [2, 3]. In addition, when left ventricular dysfunction is objectified there is the notion that viability is rare and intervention therefore futile. Conversely, multiple studies have demonstrated that viability is present in the vast majority of CTO patients and revascularisation should not be withheld [4]. That being said, some will allude to the lack of prognostic benefit in the few thus far conducted randomised clinical trials (RCTs). This is certainly not disputed, but holds true for all RCTs conducted in patients with stable CAD pertaining to hard endpoints like myocardial infarction and (cardiac) death [5]. It is becoming increasingly apparent that revascularisation in this subset of patients should focus on alleviating symptoms, enhancing quality of life, and reducing the number of anti-anginal drugs taken daily if a CTO is left untreated [6]. In this respect, PCI CTO is no different from regular PCI.

The second misconception is based on historical data that showed PCI CTO to be accompanied by low success and high complication rates, questioning the validity of this mechanical revascularisation procedure because the risk/benefit ratio may be considered unfavourable. In contemporary practice, however, the landscape has changed through the implementation of modern strategic and technical advancements. In luminary centres, the success rate has reached around 90% in unselected patients with acceptable rates of complications comparable to those observed in other forms of complex PCI [7]. So why is PCI CTO still not offered to all of our eligible patients? The answer is simple: it is difficult! If we cannot achieve success within a given skill set, sometimes clinical indications seem to blur and alternative treatment strategies become more acceptable (like CABG and OMT), thereby maintaining the status quo. The solution to this problem is simple in its complexity. We need to learn …

This issue of the *Netherlands Heart Journal* is focused on CTO and contains compelling articles on a variety of topics within the field. van Veelen et al. provide insight into the incidence and outcome of PCI CTO specifically in the Netherlands and present a meta-analysis of currently available RCTs [8, 9]. Physiological consequences of CTOs are discussed by Keulards and colleagues, including the impact of intervention, whereas van de Werf et al. describe the temporal evolution and characteristics of a single-centre experience [10, 11]. The most under-utilised technique of PCI CTO in the form of antegrade dissection re-entry is highlighted by Berkhout et al. [12]. Non-invasive planning of PCI CTO with computed tomography is subsequently debated by Opolski and colleagues [13]. Finally, a pivotal framework of complication management is summarised by Karacsonyi et al. [14]. Although all of these articles help us to propel the field forward, the fundamentals deserve attention before PCI centres can engage in such endeavours. Developing a PCI CTO programme is relatively straightforward, yet it requires years of dedication and training to mature. It is of the utmost importance that potential operators realise that recanalisation of a CTO encompasses antegrade as well as retrograde wiring, and dissection/re-entry techniques. Even though it is beyond the scope of this editorial to provide extensive insights into the development of such as programme, a few fundamental steps can briefly be summarised.

Operators should not be novices and should be proficient in the basics of complex procedures that deal with, for example, left main disease, bifurcation treatment, utilisation of guide extensions, rotational atherectomy, and complication management. It is strongly recommended that such programmes rely on at least two dedicated operators who ‘double scrub’, as many factors come into play that are not easily managed by one operator alone. Upon initiation, the first and foremost prerequisite for virtually all procedures is double arterial access (i.e. engaging the right and left coronary artery simultaneously). Whatever strategy is employed, double access is mandatory to completely visualise the proximal cap, lesion length, distal target, and collaterals. Once double access is ensured, antegrade wire manipulation with the use of a microcatheter needs to be mastered. After this stage, large registries have demonstrated that in unselected patients roughly half of these attempts will be unsuccessful [15]. The J‑CTO score is highly predictive for this purpose [16]. The most common alternative strategy to succeed is a retrograde approach, whereby septal and epicardial collaterals, as well as bypass grafts, can act as conduits. It needs to be emphasised that while retrograde access can be achieved relatively easily, in the majority of cases these procedures end up as dissection techniques (i.e. reverse CART) to open the artery. All in all, approximately one-third of patients are treated retrogradely in tertiary referral centres. However, sometimes antegrade dissection re-entry techniques are required when wiring fails and retrograde access is not available or considered too complex. Dedicated equipment is available and in the hands of skilled operators can be quite successful.

In interventional cardiology, PCI CTO is often referred to as the final frontier. Unfortunately, its dissemination is not widespread, and there is an unmet need for many patients who remain symptomatic after declined or failed attempts. We need to realise that this kind of treatment should not be considered a standard of care in any random PCI centre, nor should it be expected to be. It is more prudent to allocate specialised care to experienced centres. Paraphrasing Craig Thompson, one of the founding fathers of PCI CTO, we should shift the paradigm and not ask ourselves why the vessel should be opened, but what the justification is for leaving it closed [17].
