# Correction to: Percutaneous coronary intervention for chronic total coronary occlusion: Do. Or do not. There is no try

**DOI:** 10.1007/s12471-020-01535-6

**Published:** 2020-12-18

**Authors:** P. Knaapen, J. P. Henriques, A. Nap, F. Arslan

**Affiliations:** 1 Department of Cardiology, Heart Center, Amsterdam UMC, University of Amsterdam, Amsterdam Cardiovascular Sciences, Amsterdam, The Netherlands; 2 Department of Cardiology, Vivantes Klinikum am Urban, Berlin, Germany

**Correction to:**

**Neth Heart J 2020**

10.1007/s12471-020-01531-w

The original version of this article unfortunately contained a mistake. The title was incomplete; the corrected title is given above. The original article has been corrected.

